# Effects of size scaling on cellular dynamics and tissue patterning in tooth root organoids

**DOI:** 10.3389/fbioe.2026.1801110

**Published:** 2026-08-07

**Authors:** Tia C. Calabrese, Kristi Rothermund, Fatima N. Syed

**Affiliations:** 1 Department of Bioengineering, Swanson School of Engineering, University of Pittsburgh, Pittsburgh, PA, United States; 2 Center for Craniofacial Regeneration, School of Dental Medicine, University of Pittsburgh, Pittsburgh, PA, United States; 3 Department of Oral and Craniofacial Sciences, School of Dental Medicine, University of Pittsburgh, Pittsburgh, PA, United States; 4 McGowan Institute for Regenerative Medicine, Pittsburgh, PA, United States

**Keywords:** cellular dynamics, dental pulp stem cells, periodontal ligament stem cells (PDLC), scaffold-free 3D culture, tissue patterning

## Abstract

Organoids offer a novel platform to study developmental and reparative processes and hold promise as model systems for drug discovery and personalized medicine. To maximize their utility, we investigated the capacity of organoids, specifically tooth root organoids, to be scaled down in size. We previously established that dental pulp stem/progenitor cells (DPSCs) and periodontal ligament (PDL) stem/progenitor cells (PDLSCs) co-cultured using scaffold-free tissue engineering approaches, self-assemble into tooth root organoids with anatomically organized layers of pulp-, dentin-, cementum-, and PDL-like tissues. These organoids were originally formed in 6 well plates. To test the effects of scaling down construct size, DPSC-PDLSC constructs were now generated with fixed initial cell density in 6-, 12-, and 24-well plates. While construct diameter initially scaled linearly with culture surface area, over time samples generated in the larger wells condensed more than their smaller counterparts resulting in final construct sizes which deviated from the initial linear relationship. Furthermore, this differential size decrease coincided with altered mineralized tissue patterning, where samples generated in the smaller dishes had greater relative mineral deposition that lacked predictable patterning unlike the distinct anatomically patterned mineralized and soft tissue layers seen in constructs formed in the larger wells. Interestingly, samples formed in the smaller wells had increased apoptotic activity at the periphery, suggesting that cellular dynamics vary with initial construct size. These findings demonstrate that reducing initial cell number in scaffold-free DPSC-PDLSC constructs alters cellular behavior and tissue architecture, suggesting the existence of a critical cell mass required for consistent and proper tooth root organoid formation.

## 1 Introduction

Organoids bridge the gap between studies that utilize simple two-dimensional (2D) cell culture and complex *in vivo* models (Langhans, 2018; Lancaster and Huch, 2019; Liu et al., 2021). They provide a platform to controllably study human cellular processes and interactions within organized, multi-tissue systems that more closely mimic natural structures than 2D cell culture and minimize the potential confounding factors of animal studies (Hofer and Lutolf, 2021; Rossi et al., 2018). These specialized three-dimensional (3D) constructs have proven valuable in drug discovery, personalized medicine, regenerative therapies, and as high-fidelity models of human repair and development (Langhans, 2018; Lancaster and Huch, 2019). Determining the capacity of organoids to be scaled down in size could optimize their utility for a variety of applications. In personalized medicine, this fine-tuning would allow for the generation of the maximum number of organoids from donated tissue increasing treatment testing capacity. Additionally, drug discovery often demands high-throughput formats, which would ideally utilize the smallest possible organoids to improve efficiency and reduce resource consumption (Langhans, 2018).

Discussions of organoid scaling often focus on limitations of maintaining large constructs in culture, citing the formation of necrotic cores due to diminished oxygen and nutrient diffusion into the tissue compounded with the failure to remove cytotoxic waste products (Nwokoye and Abilez, 2024). However, scaling construct size down also poses biological challenges. Given the importance of signaling gradients in organoid patterning (Koh and Hagiwara, 2023; Zheng and Loh, 2022), altering organoid size could affect tissue patterning by shifting concentrations of media components or endogenously produced morphogens across the construct. It has also been postulated in computational modelling studies that below a certain size threshold, organoids may not follow physiologic allometric scaling laws with regard to metabolic factors (Ahluwalia, 2017; Magliaro et al., 2019). Namely, two factors were studied via these models: Kleiber’s Law, which relates an organism’s body size to its basal metabolic rate, and the oxygen consumption at a given size. While specific size ranges differ dependent upon materials used to form these organoids, these mathematical models predicted a narrow range of sizes within which organoids, specifically brain organoids, would both follow Kleiber’s Law and maintain a viable core. Alterations in metabolic behavior could have strong implications for cell behavior and the physiologic relevancy of these novel organoids (Ahluwalia, 2017; Magliaro et al., 2019).

A critical first step in investigating the capacity to scale down organoid size is the identification of an appropriate model system. Teeth are often cited as a useful model to study general tissue assembly and reparative processes that could be applied to systems throughout the body due to their variety of interfacing biochemically and structurally distinct layers of mineralized and soft tissues (Pagella et al., 2020). The core of the tooth is the dental pulp, a vascularized and innervated soft tissue critical for the survival of the tooth and for relaying sensory cues. The pulp interacts heavily with dentin, the surrounding mineralized tissue which supports the bulk structure of the tooth. In the root region, dentin is surrounded by cementum, a second mineralized tissue which works with the periodontal ligament (PDL) to anchor the tooth to the jaw. In the crown region, the dentin is enclosed within the hardest tissue in the body known as enamel which withstands the large forces of mastication (Nanci, 2013). Several postnatal stem/progenitor cell populations have been identified in the tooth, including dental pulp stem/progenitor cells (DPSCs) (Gronthos et al., 2000) and periodontal ligament stem/progenitor cells (PDLSCs) (Seo et al., 2004). These cells naturally mediate dental tissue homeostasis and repair (Nanci, 2013; Gronthos et al., 2000; Seo et al., 2004; Goldberg and Smith, 2004; Goldberg et al., 2011; Korkmaz et al., 2021; Goriuc et al., 2023). Using scaffold-free tissue engineering approaches, we have established that DPSCs and PDLSCs are capable of self-assembling into organoids that resemble the dentin-pulp complex and PDL-cementum, respectively (Rothermund et al., 2022; Basu et al., 2019; Syed-Picard et al., 2014; Syed-Picard et al., 2013). Furthermore, we have recently found that when co-cultured in scaffold-free constructs, DPSCs and PDLSCs form higher-order full tooth root organoids that contain distinct, anatomically organized and biochemically distinct layers resembling the dental pulp, dentin, cementum, and PDL-like tissues (Calabrese et al., 2023). In the tooth specifically, during development it is known that the tooth germ size and relative proportions of the dental epithelium and mesenchyme can impact developmental timeframes and key morphological features (Kavan et al., 2007). Therefore, the investigation of the scalability of these particular organoids can also lend critical insights into the biological mechanisms at play in their formation. Additionally, given their scaffold-free and highly biomimetic nature, results from this study may be further extrapolated to draw hypotheses about mechanistic features within the native tissue.

In this study, we investigate the effect of size on tooth root organoid patterning by creating constructs of varying dimensions from the same initial cell density. This approach aims to uncover whether a critical cell mass is necessary for proper tissue patterning, with implications for organoid use in high-throughput settings such as pharmaceutical testing and personalized medicine, and the elucidation of cellular mechanisms involved in organoid formation processes.

## 2 Methods

### 2.1 Stem/progenitor cell isolation

Healthy human third molars from a 16-year-old female were collected from the University of Pittsburgh School of Dental Medicine after routine extraction (University of Pittsburgh Institutional Review Board STUDY21040099). Stem/progenitor cells were isolated following previously established procedures (Gronthos et al., 2000; Seo et al., 2004). Briefly, molars were washed in phosphate-buffered saline (PBS) (Fisher Scientific) supplemented with 2X penicillin and streptomycin (P/S) (Fisher Scientific). PDL tissue was scraped from the middle third of the root using a scalpel in order to avoid the root apex or the collection of any gingival tissue. Then, the tooth was mechanically opened and pulp tissue was extracted using forceps. Tissues were separately minced to pieces of approximately 1 mm, then digested in 3 mg/mL collagenase (EMD Millipore) + 4 mg/mL dispase (EMD Millipore) at 37 °C for 1 h, with vortexing approximately every 15 min to ensure complete digestion of the tissue. Tissue digests were passed through a 70 µm strainer to obtain a single cell solution. Cells were plated at a density below 10,000 cells/cm^2^ in growth media containing Dulbecco’s Modified Eagle Medium (DMEM) (Gibco), 1X P/S, and 20% fetal bovine serum (FBS) (R&D Systems). Colonies were expanded, then cryogenically frozen for use between passages 2 and 5.

### 2.2 Construct formation

Constructs were formed using methods similar to those previously described (Rothermund et al., 2022; Basu et al., 2019; Syed-Picard et al., 2014; Syed-Picard et al., 2013; Calabrese et al., 2023; Syed-Picard et al., 2009). Briefly, culture wells of 6-well (9.6 cm^2^ culture area/well), 12-well (3.85 cm^2^ culture area/well), and 24-well (1.93 cm^2^ culture area/well) plates were filled with 2, 1, and 0.5 mL of polydimethylsiloxane (PDMS) (Dow), respectively, according to manufacturer’s instructions, then coated with 3 μg/cm^2^ laminin (APExBIO). Plates were dried overnight in a tissue culture hood, then rinsed in PBS and UV-sterilized for 1 h before incubating with growth media for a minimum of 24 h before use in culture.

Culture conditions were proportionally scaled such that the relative dimensions of all external factors were preserved across experimental groups. To form scaffold-free constructs, DPSCs and PDLSCs were mixed in a 1:1 ratio and plated onto the previously coated plates at a density of approximately 20,000 cells/cm^2^ in growth media, yielding a total of approximately 200,000 cells, 80,000 cells, and 40,000 cells/well for the 6-, 12- and 24- well plates, respectively. Media volume was scaled to dish size at 0.2–0.3 mL/cm^2^. After 2 days, media was switched to a differentiation medium (DM1) containing the same components as the growth media with the addition of 50 μg/mL ascorbic acid (Sigma-Aldrich), 50 mM β-glycerophosphate (Fisher Scientific), 10 nM dexamethasone (Sigma-Aldrich), and 5 ng/mL fibroblast growth factor-2 (PeproTech). Three days after the addition of DM1 when cells reached confluence, media was switched to a final differentiation medium (DM2) containing the same components as DM1 with serum concentration reduced to 5% and with the addition of 2 ng/mL transforming growth factor β-1 (TGFβ-1) (Peprotech). Cell sheets then naturally contracted to form a 3D construct within 24 h of the addition of DM2 and were kept in DM2 for a total of 14 days. Samples were fixed overnight in 10% formalin (Fisher Scientific) at 4 °C for histological analysis.

### 2.3 Histological analysis

Constructs were embedded in Optimal Cutting Temperature (OCT) Media (Fisher Scientific), then sectioned using a cryostat at a thickness of 5 µm. To evaluate gross tissue morphology, hematoxylin 1 and eosin Y (H&E) (Fisher Scientific) staining was performed. Both 2% alizarin red S (ARS) (Sigma-Aldrich) using standard methodology and Von Kossa staining (Calcium stain kit; Scytek Laboratories) following manufacturer instructions were performed to evaluate mineral deposition. This histological staining was repeated on sections acquired from separate constructs: n = 6 24-well samples, n = 6 12-well samples, and n = 4 6-well samples. Sample sizes varied due to the exclusion of samples due to technical challenges in acquiring histological sections suitable for analysis. Immunohistochemistry was performed on representative tissue sections for n = 3 6-well, n = 3 12-well, and n = 4 24-well samples against primary antibodies for proliferating cell nuclear antigen (PCNA) (Novus Biologicals) and cleaved caspase-3 (CC3) (Novus Biologicals) to evaluate cell proliferation and apoptosis, respectively; both antibodies were used at a 1:50 dilution. Fluorescently tagged secondary antibodies Alexa Fluor goat anti-rabbit 647 (Invitrogen) and Alexa Fluor goat anti-mouse 647 (Invitrogen) were used to visualize signal at a dilution of 1:500. Sample numbers differed here as well due to challenges during sectioning, as well as a reduced number of available sections from the center of each sample, which was necessary to make accurate comparisons across groups. Negative controls were performed by omitting primary antibody. Images were acquired on a Nikon Eclipse Ti2-E spinning disk confocal.

### 2.4 Image quantification

Images of constructs were taken on a Leica S9D stereoscope of 2, 6, and 14 days after formation. Using ImageJ, the circumference of each construct was measured, then converted to diameter assuming an approximately spherical shape. At day 2, n = 4 24-well, n = 3 12-well, and n = 5 6-well samples were measured. At day 6, n = 6 24-well samples, n = 5 12-well, and n = 5 6-well samples were measured. At day 14, n = 7 24-well, n = 10 12-well, and n = 5 6-well samples were measured. Construct diameter was plotted against culture surface area over time and linearly fitted, and line slopes and *R* ^2^ values were calculated using Microsoft Excel. Construct diameter versus time was plotted separately for each experimental group; the slopes of these lines between each time point were also determined (Microsoft Excel).

To quantitatively evaluate localized PCNA and CC3 expression, fluorescent intensity measurements were taken from the edge and the center of each section on both immunostained samples and respective negative controls. In the prior study which established these tooth root organoid systems, the localization of PDLSCs to the edge of the construct and DPSCs to the center of the construct were established and quantified. This quantification showed that the outer 26% of the section by radius was made up of PDLSCs and the remaining 74% central region comprised DPSCs (Calabrese et al., 2023). Here, the same percentiles were used to estimate the edge and center of each tissue. Namely, the circumference of each section was measured and diameter was extrapolated, square regions of interest (ROI) were generated where the length of each side of the square equaled 26% of the radius. The size of the ROI was kept consistent for the measurements of the center of the tissue in order to avoid discrepancies in variation across a region. Intensity measurements were taken from 4 ROIs from the edge and center regions of the section, respectively. Intensity measurements were also taken from outside of the constructs as background. Background measurements were subtracted from edge and center measurements, respectively. Intensity measurements were normalized to those acquired from the negative control. Edge measurements were calculated relative to the center of the sample and plotted as mean +/- standard deviation. Differences between groups were evaluated using a one-way ANOVA with Tukey HSD *post hoc* testing in Minitab Statistical Software where p < 0.05 was considered statistically significant.

To determine relative mineral amount and distribution, histological sections acquired from the center of the constructs were stained with Alizarin Red S. Quantitative image analysis was performed by first digitally cropping out regions outside of the histological section to isolate the section without interference from shadow or debris in the total image field (Microsoft PowerPoint using the “remove background” feature). Using ImageJ, 3 threshold values ranges were set in grayscale images: full section including any holes resulting from natural pores in the sample to determine the overall shape of the construct (0–210), section excluding any pores (0–195), and mineralized pixels (0–130). Thresholds were defined manually in ImageJ based on 3 representative samples before mass processing. Then, all images were imported into MATLAB, where radial concentric regions of the sections were defined in increments of 5% by distance from the center. The proportion of mineralized tissue to total tissue within each concentric region was determined and plotted as a distribution. Statistical differences in distributions were determined using pairwise Kolmogorov-Smirnov tests with a Bonferroni correction such that differences with p < 0.01 were considered significant; this analysis was performed using Minitab Statistical Software. Additionally, gross percentage of mineral was calculated as a ratio of the mineralized pixels to the total pixels of the entire section. To evaluate statistical differences in total mineral, a one-way ANOVA was performed with a Tukey HSD *post hoc* test in Minitab Statistical Software where p < 0.05 was considered statistically significant.

## 3 Results

### 3.1 Scaffold-free DPSC-PDLSC constructs formed in different size well plates scale in size initially, but size scaling becomes dysregulated over time

**FIGURE 1:**
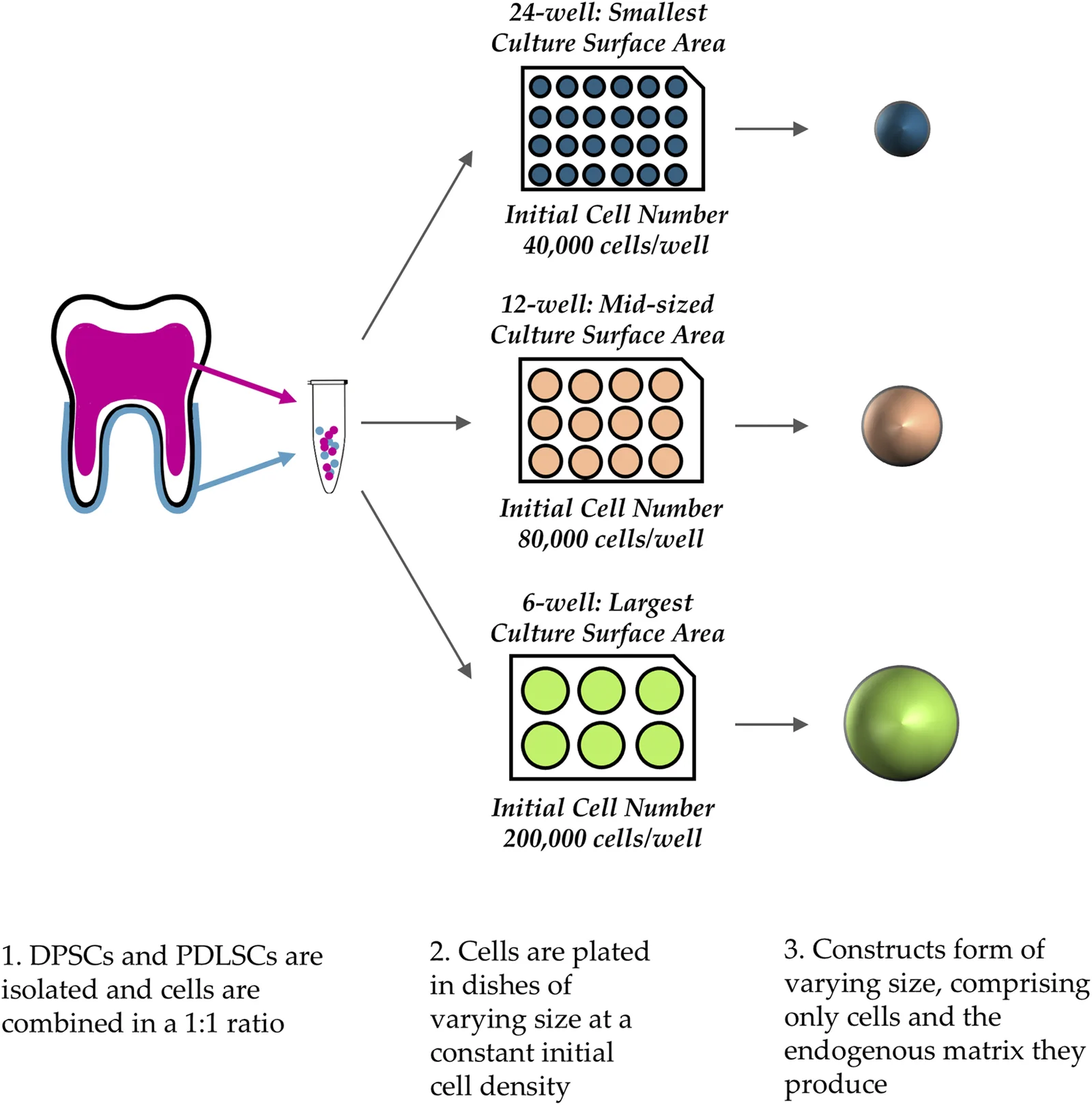
Schematic of DPSC-PDLSC construct formation. Scaffold-free constructs were formed using a 1:1 co-culture of human DPSCs and PDLSCs. The capacity of constructs to scale in size was assessed by forming the constructs in different size well plates (24-well, 12-well, and 6-well).

**FIGURE 2:**
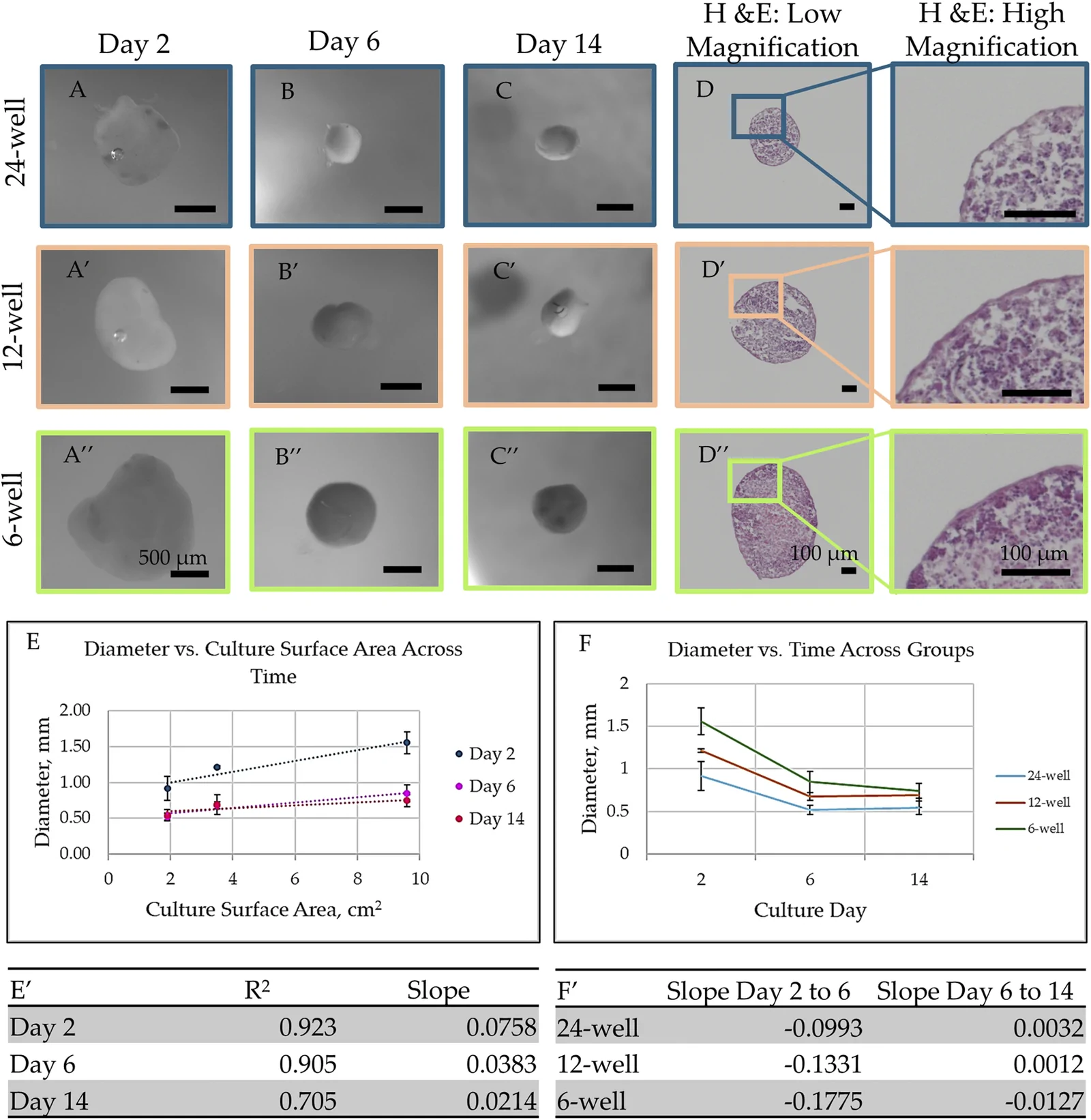
Organoid sizes over time. **(A-C’’)** Stereoscope images of constructs were taken at days 2 **(A, A’, A’’)**, 6 **(B, B’, B’’)**, and 14 **(C, C’, C’’)** after construct formation. **(D-D’’)** Hematoxylin and Eosin staining (H and E) showed that all constructs were solid and cellular throughout after 14 days of culture. **(E)** Quantification of construct diameter vs. culture surface area showed that constructs initially scale linearly, but over time lose adherence to linearity. **(E’)** Table listing slopes and coefficient of determination (R^2^) of lines plotted in E. **(F)** Plotting construct diameter vs. time revealed that this loss final construct size linearity was due to size decrease of 6-well samples between days 6 and 14, while 12-well and 24-well samples did not change in size. **(F’)** Table listing slopes of lines plotted in F. Scale bars **(A–C”)** = 500 µm. Scale bars **(D-D”)** = 100 µm.

Scaffold-free DPSC-PDLSC constructs were generated in different culture well sizes to determine whether the size of tooth root organoids can be controlled via limiting the area of initial cell growth (Figure 1). The cells were plated onto the treated well dishes and allowed to form a cell sheet that subsequently detached from the substrate and contracted into a 3D structure. Two days after formation, constructs formed in different size wells were notably different in size, but these size differences become less evident at days 6 and 14 (Figures 2A–C”). These shifts in relative construct size over time were confirmed with quantitative image analysis (Figures 2E,E’, F,F’). At 2 days after formation, there is a strongly linear relationship between construct size and culture surface area as seen by the relatively steep slope of the line plotting these parameters and the high coefficient of determination (R^2^); however, at day 6 and 14, the slopes of these lines substantially flatten and a linear trendline becomes much less well-fit to the data (Figure 2E’). Plotting construct diameter versus time (Figure 2F) revealed that samples within all groups undergo a substantial shift in construct size reduction between days 2 and 6; however, the sharpest reduction in size occurs in samples formed in the 6-well plates. Furthermore, between days 6 and 14, 6-well samples continue to decrease in size while the size of the smaller 12-well and 24-well samples remain constant or even slightly increase (Figure 2F’). This unequal rate of construct shrinkage explains why the diameter of the samples across groups does not scale linearly by day 14. These inherent differences in size reduction across experimental conditions imply that initial cell mass affects intrinsic cellular dynamics within these engineered constructs.

Hematoxylin and eosin (H&E) staining was performed on the constructs 14 days after formation. All of the constructs appear similarly solid and cellular throughout (Figure 2D’). Similar to what was observed previously in tooth root organoids formed in wells of 6-well plates (Calabrese et al., 2023), the center regions of all of the constructs have a rounded cell morphology. Furthermore, a distinct peripheral tissue structure is seen on all samples where the cells and extracellular matrix appear more elongated; this indicates that a simple bilayered structural patterning is formed and maintained in all samples regardless of construct size.

### 3.2 Localized apoptotic activity is induced by modulating scaffold-free DPSC-PDLSC construct size

**FIGURE 3:**
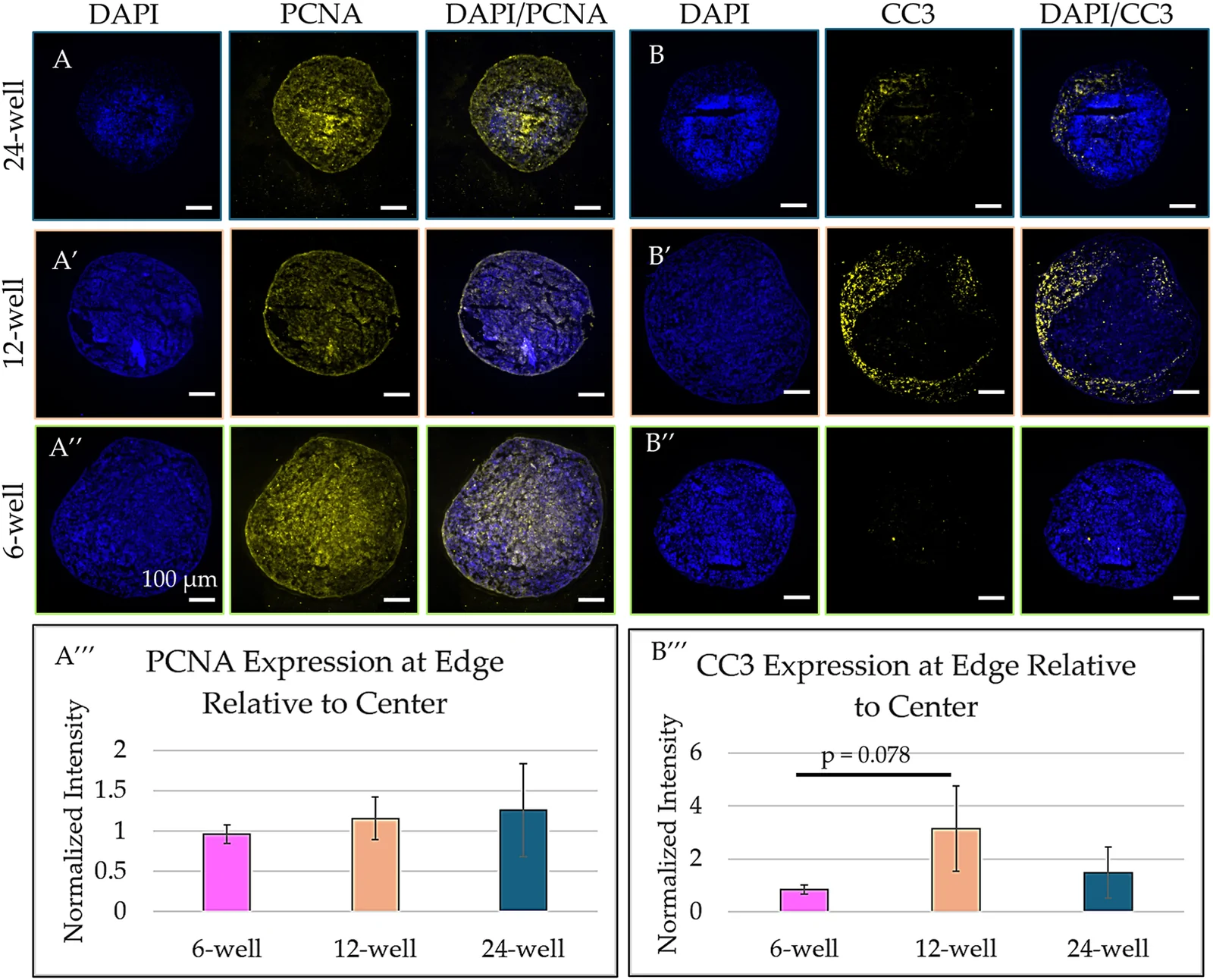
Expression of proliferation and apoptosis markers. **(A-A’’)** Immunostaining for proliferating cell nuclear antigen (PCNA; yellow) showed that all constructs displayed cellular activity throughout the tissue for the duration of culture. **(A’’’)** Quantitative analysis of PCNA expression in the peripheral relative to central regions of constructs formed in wells of different sizes. **(B-B’’)** Staining for cleaved caspase-3 (CC3; yellow), a marker of apoptosis, showed that 6-well constructs **(B’’)** exhibited little to no apoptotic activity. In contrast, 24-well **(B’)** and 12-well **(B’’)** samples showed a trend towards an increase in apoptosis at the periphery of the construct. **(B’’’)** Quantitative analysis of CC3 expression in the peripheral relative to central regions of constructs formed in wells of different sizes. Scale bars = 100 μm; DAPI staining used to detect nuclei (blue) in **(A-A”)** and **(B-B”)**.

To determine whether shifts in proliferative and apoptotic activities contribute to the changes in construct size observed over time, histological sections were immunofluorescently stained for proliferating cell nuclear antigen (PCNA) and cleaved caspase-3 (CC3), respectively. PCNA activity was observed throughout constructs formed in all well sizes (Figures 3A-A′′′), indicating positive cellular activity throughout all samples. Minimal CC3 expression, a marker of apoptosis, was detected in the constructs formed in the 6-well plates. Interestingly, increased expression of CC3 expression was found in peripheral regions of the constructs formed in the 12 and 24-well plates (Figures 3B-B′′′). Notably, although it warrants further investigation, this apoptotic activity is likely not the cause of the size reductions observed in the constructs over time since the samples formed in the 6-well plates exhibited the greatest reduction in size over time but lacked apoptotic cell activity. Alternatively, this could suggest that the construct size reduction over time could be driven more by condensation-like mechanical contractions. These potential differences in mechanobiology could strongly alter many cellular processes including cell differentiation or cell-cell communication.

### 3.3 Tissue patterning becomes dysregulated in scaffold-free DPSC-PDLSC constructs formed in smaller-sized wells

**FIGURE 4:**
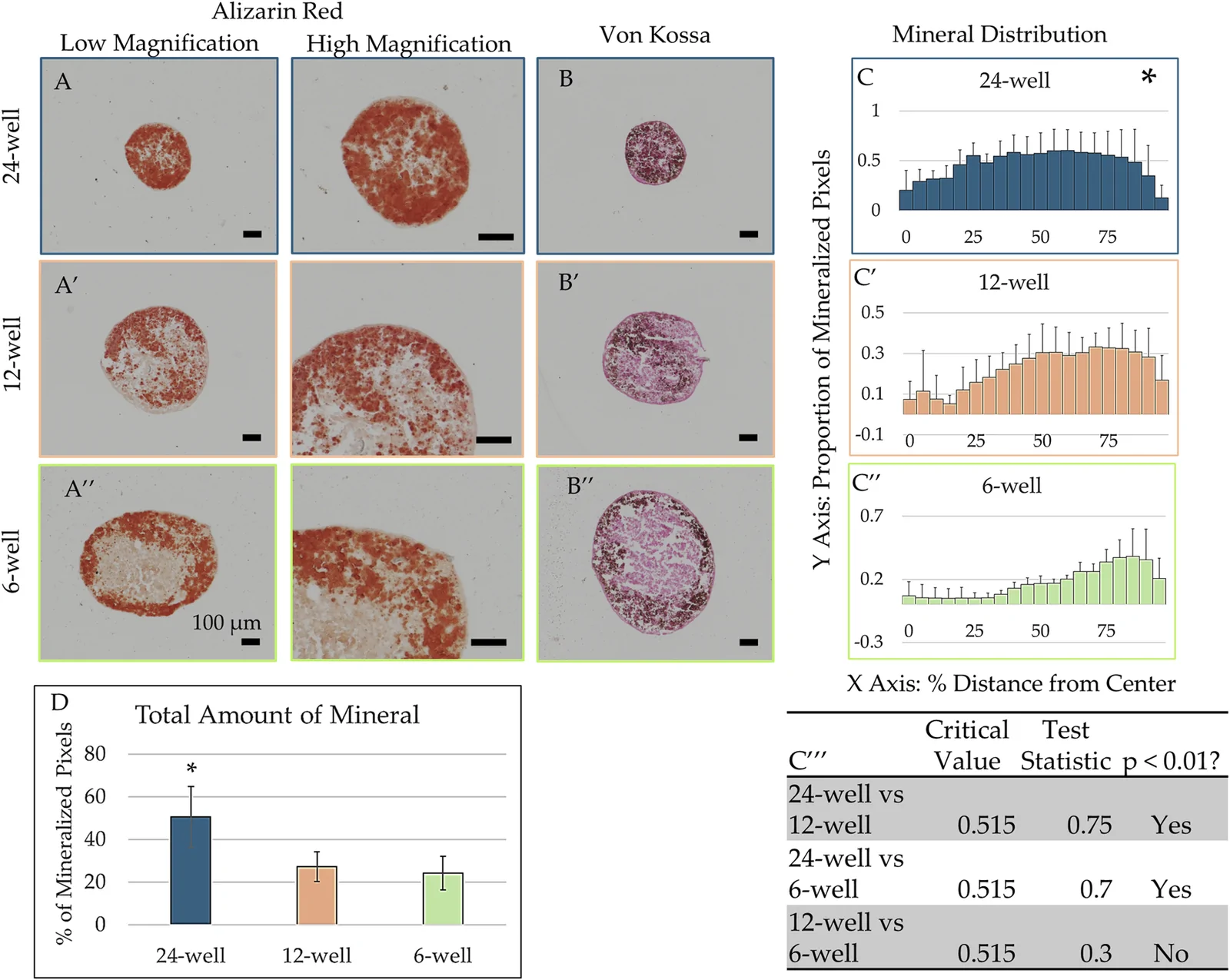
Mineral patterning in DPSC-PDLSC constructs of different sizes Alizarin Red S staining **(A-A’’)** and Von Kossa **(B-B”)** staining showed positive calcium and phosphate deposits in all samples. Positive Alizarin Red S and Von Kossa staining patterns matched indicating that positive staining represents mineral deposition. **(C-C’’)** Quantitative analysis of mineralized tissue patterning is presented as histograms showing the proportion of mineral-positive tissue as a function of distance from the construct center; 6-well samples exhibit a tooth root-like mineral pattern with a central soft tissue, mineralized layer, and soft peripheral tissue, this distribution flattens out in smaller samples and becomes significantly different in the smallest 24-well samples. **(C’’’)** Table showing statistical differences among the distributions shown in **C-C’’**. **(D)** Gross mineral quantification showed that 24-well samples have a significantly higher total relative amount of mineral than 12-well and 6-well counterparts (p = 0.003). Asterisks show groups with statistical differences. Scale bars = 100 µm.

Both alizarin red S (ARS) staining and Von Kossa staining, which detect calcium deposition and phosphate, respectively, were performed to evaluate mineral deposition (Figures 4A-B’’) in the scaffold-free DPSC-PDLSC constructs. Von Kossa staining was used to confirm that ARS results reflected mineralization and not cell death, since dying cells release intercellular calcium; ARS and Von Kossa staining patterns closely matched, indicating that ARS staining did accurately reflect the mineral pattern of the tissues. Consistent with our prior work (Calabrese et al., 2023), constructs formed in 6-well plates exhibited a tooth root like mineral pattern, with a central unmineralized tissue, where a soft pulp-like tissue would be localized, surrounded by a mineralized structure, where dentin and cementum would be localized, that is enclosed within a second outer unmineralized tissue, like an outer PDL. However, this patterning became dysregulated in the smaller constructs formed in the 12- and 24-well plates; the outer unmineralized tissue is present in all samples however, mineral deposition becomes more evenly dispersed throughout the center (Figures 4A-B”). Quantitative analysis of the mineralization profile from the center to the edge of the constructs substantiated these shifts in mineralized tissue patterning in constructs formed in different size wells (Figures 4C,C’, C”). A statistically significant difference in this mineral patterning was detected in the 24-well samples (Figure 4C’”). Furthermore, a significant increase in total mineral deposition per section area was observed in 24-well samples (Figure 4D), which coincides with its disruption of tooth root-like mineral patterning. This indicates tooth root organoids cannot be scaled down in size by forming DPSC-PDLSC scaffold-free constructs in smaller dishes.

## 4 Discussion

Primitive tissue mass and intrinsic mechanobiology play critical roles in embryonic organogenesis (Sui et al., 2023; Mammoto et al., 2015; Hashmi et al., 2014; Mammoto et al., 2011; Cai et al., 2007). This work establishes that these components of natural development are also necessary for proper patterning of tooth root organoids. Here, we show that a minimum initial cell number threshold must be met to consistently drive scaffold-free DPSC-PDLSC constructs to self-organize into tooth root organoids. Reducing the initial cell number results in a clear dysregulation of tissue patterning. Additionally, scaffold-free DPSC-PDLSC constructs undergo a size reduction over time, and both the magnitude and rate of this size decrease are dependent on the initial construct size. Differences in construct compaction coincide with marked changes in cell fate. These findings provide new, foundational insight on mechanisms orchestrating tissue patterning in tooth root organoids.

Organoids present a unique opportunity to methodically study human cellular behavior in a physiologically relevant 3D model system. In this work, organoids were generated using scaffold-free tissue engineering approaches that promote cells to deposit and organize endogenous extracellular matrix (ECM) into a 3D structure. These constructs bypass the external stimuli introduced by exogenous scaffold materials and preserve natural cell-cell and cell-ECM interactions (Lee et al., 2017) making them particularly well-suited for studying cellular mechanisms underlying development and repair. Members of our lab have also engineered organoids that emulate bone-periosteum (Syed-Picard et al., 2009), PDL-cementum (Basu et al., 2019), and the dentin-pulp complex (Rothermund et al., 2022; Syed-Picard et al., 2014; Syed-Picard et al., 2013). Importantly, each of these systems were formed using identical culture methods, differing only in their cell source (bone marrow stromal cells, DPSCs, and PDLSCs, respectively); this indicates that these stem/progenitor cells are intrinsically programmed to drive the assembly of organoids that resemble the organ systems from which they were derived. This further emphasizes that scaffold-free systems can more closely mimic native biology, and thus studying the effects of scaling using these systems can yield key insights into mechanisms of tissue patterning.

Initially following formation, scaffold-free DPSC-PDLSC construct size scaled linearly with the surface area on which the cells were plated; however, this linear size scaling is lost with time. The formation of these constructs entails forming cell sheets that contract into a 3D structure. The size of the naive constructs is likely dictated by the cell culture area on which the cell sheets were formed where smaller wells produced smaller sheets. At early stages of culture, all of the constructs exhibited a size reduction (day 2-day 6). Additionally, the magnitude of the change is size-dependent, with larger constructs exhibiting a more significant decrease in size. However, at later stages only the constructs generated in the wells of 6 well plates continued reducing in size whereas constructs formed in the smaller dishes did not (day 6-day 14). These divergent trends suggest potential size-dependent shifts in cellular behavior and altered mechanical microenvironments in constructs formed with differing initial mass. Future studies will focus on directly measuring any potential condensation events.

Differences in size change over time of DPSC-PDLSC constructs formed in the varying size well dishes corresponded with distinct changes in cellular activity and tissue patterning. A significant difference in mineralized tissue localization was observed across constructs of different size. The largest, 6-well constructs displayed mineral pattern that emulated the natural pattern of the tooth root whereby the central pulp is unmineralized, surrounded by mineralized layers of dentin and cementum, and enclosed in the soft PDL. In contrast, the smallest samples (24-well) exhibited the greatest relative amount of mineral deposition, and the mineral patterning was diffusely distributed across the constructs failing to consistently reproduce organized tooth root-like architecture. This could potentially reflect increased diffusion of osteogenic components into the smaller constructs. Although a significant difference in the mineral pattern of constructs formed in the 12-well and 6-well samples was not detected, the mineral patterning of the samples formed in the 12-well plate was less distinctly organized than that in 6-well samples as detected by flattened shape of histograms of mineral profile. In pursuit of optimizing organoid size for scalability during drug discovery and personalized medicine, these data suggest that there is some flexibility to decrease construct size before patterning is disrupted. It is possible that the rate of contraction may offer some insight in this regard, given the key role of mechanical influences during tooth development (Sui et al., 2023; Mammoto et al., 2015; Hashmi et al., 2014; Mammoto et al., 2011).

The observation that the larger DPSC-PDLSC constructs, formed in the 6-well plates, lacked apoptotic activity but exhibited the greatest relative reduction in size over time suggests that their shrinkage may have resulted from developmentally-mimetic condensation events. At early stages of natural tooth development, condensation occurs in the papilla, the tissue which gives rise the dentin-pulp complex, and concludes in the late bell stage with the onset of odontogenic differentiation; in contrast, condensation of the follicle, which forms the cementum and PDL, continues through root elongation until Hertwig’s Epithelial Root Sheath, a signaling tissue located between the papilla and the follicle and critical to root formation, dissociates allowing the follicular cells to come into contact with dentin, marking the onset of cementogenic differentiation (Sui et al., 2023). Condensation coincides with the production of specific signaling molecules, as well as significant compressive forces exerted on cells in a gradient-dependent manner, which may similarly play a role in tissue patterning of these organoids (Sui et al., 2023). Since the DPSCs and PDLSCs utilized for organoid formation here are more commonly considered in terms of their role in homeostatic and reparative processes and not development, this could suggest that DPSCs and PDLSCs involved in organoid formation may retain latent developmental programs from their respective precursors in the papilla and follicle; this speculation warrants further investigation. Alternatively, this could also imply that tissue condensation could also be a key factor in dental tissue repair. In contrast, smaller constructs lacked this condensation behavior at later culture stages and showed peripheral cell apoptosis. In tooth germ models, it has been demonstrated that odontogenesis can be recovered by the artificial application of compressive forces (Mammoto et al., 2015; Hashmi et al., 2014; Mammoto et al., 2011). Analysis of condensation markers or the activation of mechanotransduction pathways would confirm whether the tooth root organoids undergo similar condensation events seen during development. Future studies should investigate whether tissue patterning can be recovered in these smaller engineered tissues by the artificial application of compressive forces in a similar manner.

Contrary to other organoid systems where necrotic tissues are observed at depths beyond 200 μm^7^, the tooth root organoids generated here did not exhibit any signs of necrosis despite exceeding this size threshold. It is possible that due to their self-assembled and contractile nature, innate cellular mechanisms may ensure proper cellular density to maintain oxygenation across the construct. PCNA expression, a marker proliferating cells, was observed evenly across all DPSC-PDLSC constructs, confirming cellular activity across the constructs regardless of size. Interestingly, apoptotic cell death, as marked by positive CC3 signal, was only observed in smaller constructs (12- and 24 well), and localized at the periphery. Given that the same peripheral cell death was not observed in the larger 6-well samples, it is unlikely that this apoptotic activity was due to excess absolute oxygenation or signaling molecules levels at the periphery. However, differences in relative steepness of the gradient of oxygen or morphogens between the smaller and larger constructs could have altered cellular response. Also, differences in potential cell or ECM content may have contributed to the varying apoptotic behaviors in the constructs generated in the different size well plates.

While this study establishes important foundational information about the relationship between construct size and cellular behavior, some limitations within the study should be considered. Our prior work establishes the biological reproducibility of forming tooth root organoids in wells of 6-well plates (Calabrese et al., 2023). The work shown here represents constructs formed in different well-plates using cells from a single donor. While this was done intentionally to highlight that variation was an effect of scaling rather biological variability, future studies should confirm that this phenomenon is universally applicable. Additionally, the study presents observations and brings forth interesting mechanistic questions regarding cellular dynamics that may be driving these outcomes. Future work will investigate mechanisms behind the results reported here. Finally, the culture environment in this study were proportionally scaled such that absolute initial cell number, culture surface area and culture volume were all simultaneously modified to maintain relative conditions. At a system-wide level, this may impact gradients of culture media components, endogenously produced morphogens, or oxygen diffusion. Future studies should aim to individually correct for each of these parameters in order to determine whether one parameter in particular is responsible for the effects seen here, or whether it is truly a system-wide phenomenon dependent upon all changes occurring simultaneously.

This study provides key novel insights into the self-assembly mechanisms at play within tooth root organoids. We establish that scaling down constructs alters cellular dynamics, disrupts condensation, and leads to the loss of tooth root-like architecture, underscoring the importance of a critical cell mass in successful tooth root organoid formation. These ubiquitous developmental mechanisms critical for proper tooth root organoid formation may also impact the formation of organoids that emulate different organ systems throughout the body. In addition, this study provides new information suggesting that postnatal DPSC and PDLSC may recapitulate critical developmental phenomena when self-organizing into organoids, and therefore this organoid system could serve as a platform to investigate whether natural repair recapitulates development.

## Data Availability

The raw data supporting the conclusions of this article will be made available by the authors, without undue reservation.
